# Development of an Early Prediction Model for In‐Hospital Pneumonia in Adult Patients With Blunt Chest Trauma and Rib Fractures: A Clinical Risk Stratification Study

**DOI:** 10.1111/crj.70242

**Published:** 2026-11-11

**Authors:** KaiChao Yang, WeiXi Zhong, JianYin Huang, YiFeng Liu, Lei Geng, Qing Xu

**Affiliations:** ^1^ Department of Emergency Medicine Shanghai Sixth People's Hospital Affiliated to Shanghai Jiao Tong University School of Medicine Shanghai China

**Keywords:** pneumonia, prediction model, rib fractures, RibScoreblunt chest trauma, risk stratification

## Abstract

**Objective:**

Adult patients with blunt chest trauma and rib fractures are at risk for pulmonary complications. Among these, in‐hospital pneumonia may increase the need for respiratory support and adversely affect clinical outcomes. Early identification of high‐risk patients could facilitate optimization of monitoring and intervention strategies. This study aimed to construct and evaluate an early prediction model for in‐hospital pneumonia in these patients and to assess its value for risk stratification.

**Methods:**

Clinical data were retrospectively collected from consecutive adult patients with blunt chest trauma and rib fractures who were hospitalized at Shanghai Sixth People's Hospital Affiliated to Shanghai Jiao Tong University School of Medicine between March 2024 and March 2026. Candidate predictors were restricted to demographic, clinical, radiographic, and laboratory variables available at admission or within 24 h thereafter. Variable selection was performed using univariate logistic regression and least absolute shrinkage and selection operator (LASSO) regression; a multivariate logistic regression model was then constructed with due consideration of clinical interpretability. Model performance was evaluated using receiver operating characteristic (ROC) curves, calibration plots, the Brier score, bootstrap internal validation, and decision curve analysis. Risk stratification was performed according to tertiles of the predicted probability.

**Results:**

A total of 217 patients were enrolled, of whom 53 developed in‐hospital pneumonia, corresponding to an incidence of 24.4%. Multivariate logistic regression identified the following independent risk factors: age (per 10‐year increase; OR = 1.472, 95% CI: 1.148–1.920, *p* = 0.003), SpO_2_ < 95% (OR = 3.230, 95% CI: 1.523–6.939, *p* = 0.002), pulmonary contusion (OR = 3.807, 95% CI: 1.848–8.083, *p* < 0.001), hemothorax (OR = 2.921, 95% CI: 1.380–6.279, *p* = 0.005), and Abbreviated Injury Scale (AIS) ≥ 3 in other body regions (OR = 2.503, 95% CI: 1.081–5.766, *p* = 0.031). The model achieved an AUC of 0.813 (95% CI: 0.744–0.883), with a sensitivity of 75.5%, a specificity of 75.0%, and an accuracy of 75.1% at the optimal cutoff value of 0.227. Its AUC was significantly higher than that of the RibScore (0.813 vs. 0.678 [95% CI: 0.596–0.761], DeLong *p* = 0.009). The Brier score was 0.133, and the bias‐corrected AUC after 200 bootstrap resamples was 0.799. The incidence of pneumonia in the low‐, intermediate‐, and high‐risk groups was 5.5%, 18.1%, and 50.0%, respectively.

**Conclusions:**

The prediction model, which comprised age, SpO_2_ < 95%, pulmonary contusion, hemothorax, and AIS ≥ 3 in other body regions, demonstrated good discriminative ability and risk stratification value for predicting in‐hospital pneumonia in adult patients with blunt chest trauma and rib fractures, and may be used for early risk assessment upon admission.

## Graphical abstract

This single‐center retrospective study included 217 adults with blunt chest trauma and rib fractures. An early prediction model for in‐hospital pneumonia incorporated five variables available within 24 h of admission: age, SpO_2_ < 95%, pulmonary contusion, hemothorax, and extrathoracic AIS ≥ 3. The model achieved an AUC of 0.813. Observed pneumonia incidence was 5.5%, 18.1%, and 50.0% in the low‐, intermediate‐, and high‐risk groups defined by tertiles of predicted probability, respectively, supporting the model's potential for early risk stratification.

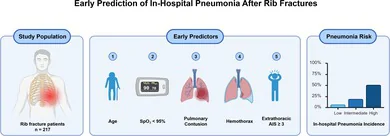

## 1 Introduction

Blunt chest trauma is a common form of injury in trauma patients, and rib fractures constitute the most frequent chest wall injury [1, 2]. Recent epidemiological data indicate that rib fractures are often accompanied by pulmonary contusion, hemothorax, pneumothorax, and injuries to other body regions. Their clinical outcomes are determined not only by the fractures themselves but also by multiple factors, including patient age, overall trauma burden, and respiratory physiologic status [3, 4]. Pain from rib fractures may restrict deep breathing and effective coughing, leading to hypoventilation, secretion retention, and atelectasis, thereby increasing the risk of pulmonary complications [5, 6]. Among these, in‐hospital pneumonia is a major complication that can further escalate the need for respiratory support, intensive care resource utilization, and adverse clinical outcomes. Elderly patients are at particularly high risk for pneumonia and other pulmonary complications because of decreased chest wall compliance, diminished respiratory reserve, and a greater comorbidity burden [7]. Classic cohort studies have also shown that age, injury severity, and rib fracture count are closely associated with pneumonia and mortality risk [8, 9].

To facilitate early risk identification, several risk assessment tools have been developed for patients with blunt chest trauma or rib fractures. RibScore, derived from chest CT findings—including the number of rib fractures, bilateral distribution, displacement, flail chest, first rib fracture, and anatomical fracture zones—has been used to predict adverse pulmonary outcomes such as pneumonia, respiratory failure, and tracheostomy [10]. Although various quantitative rib fracture scoring systems are valuable for rapid clinical assessment and communication of trauma severity, their component variables, target outcomes, and applicable populations are not entirely consistent. Some scores emphasize fracture morphology or composite pulmonary outcomes rather than specifically targeting new‐onset in‐hospital pneumonia [11, 12, 13].

As clinical prediction models are increasingly applied in emergency and trauma care, greater attention has been directed toward the external validation, calibration performance, and practical clinical utility of existing scoring tools. RibScore, an anatomical scoring system based on chest CT on admission, was originally developed to predict adverse pulmonary outcomes including pneumonia, respiratory failure, and tracheostomy, and its discriminative performance has been validated in several independent cohorts [10, 14, 15]. Subsequent studies have further suggested that the predictive performance of this score may vary across patient populations and types of concomitant injury. For example, in patients with pulmonary contusion, its discriminative ability does not consistently outperform that of other chest trauma scoring systems [13]. Some investigators have attempted to combine it with admission physiologic parameters, such as vital capacity, to more comprehensively identify patients at high risk for pulmonary complications [16]. Furthermore, in‐hospital pneumonia is influenced not only by rib fracture anatomy but also by baseline susceptibility, admission oxygenation, pulmonary parenchymal and pleural injuries, and multisystem trauma burden. Thus, relying solely on an imaging score may not fully capture an individual's pneumonia risk.

Based on these considerations, this study focused on adult patients with blunt chest trauma and rib fractures and integrated early admission data to construct an early prediction model for in‐hospital pneumonia. The model's predictive performance was evaluated in terms of discrimination, calibration, internal validation, clinical net benefit, and risk stratification, and its discriminative ability was compared with that of RibScore. The ultimate goal was to provide a reference for early identification of patients at high risk for pneumonia and for implementation of stratified management.

## 2 Materials and Methods

### 2.1 Study Design and Population

Clinical data were retrospectively collected from consecutive adult patients with blunt chest trauma and rib fractures who were hospitalized at Shanghai Sixth People's Hospital Affiliated to Shanghai Jiao Tong University School of Medicine between March 2024 and March 2026.

Inclusion criteria were: (1) age ≥ 18 years; (2) a confirmed history of blunt chest trauma; (3) at least one rib fracture documented by chest computed tomography (CT); (4) admission within 24 h after injury; (5) complete inpatient medical records and documented pneumonia outcome.

Exclusion criteria were: (1) open or penetrating chest injury; (2) preexisting pneumonia at admission or active respiratory infection prior to admission; (3) pathologic or healed rib fractures; (4) death, transfer, or discharge against medical advice within 24 h of admission, which precluded adequate outcome observation; (5) severe immunosuppression, active malignancy, or long‐term use of immunosuppressive agents; (6) pregnancy or lactation; (7) missing data for key predictor variables or outcome.

### 2.2 Data Collection and Candidate Predictors

Data were extracted by trained research staff from the hospital's electronic medical record system, laboratory information system, and picture archiving and communication system. All candidate predictors were restricted to variables available at admission or within 24 h thereafter, and the first recorded value was used preferentially to ensure that predictors preceded the development of in‐hospital pneumonia.

Candidate variables included demographic characteristics, underlying comorbidities, injury mechanism, clinical status on admission, chest injury and radiographic findings, overall trauma severity, and initial inflammatory and nutritional laboratory parameters obtained within 24 h after admission. Major variables included age, sex, body mass index, chronic comorbidities, pre‐injury use of antiplatelet or anticoagulant agents, admission SpO_2_, pain score, mechanical ventilation, rib fracture burden, pulmonary contusion, pleural injury, atelectasis, chest Abbreviated Injury Scale (AIS) score, RibScore, Injury Severity Score (ISS), white blood cell count, neutrophil‐to‐lymphocyte ratio (NLR), C‐reactive protein (CRP), procalcitonin, and serum albumin.

Continuous variables were retained on their original scale where possible. Age was modeled per 10‐year increase, and ISS was modeled per 10‐point increase. SpO_2_ was analyzed both continuously and dichotomously using a cutoff of < 95%.

### 2.3 Imaging Assessment and RibScore Calculation

All patients underwent chest computed tomography (CT) on admission as part of the standard clinical protocol. Imaging studies were independently evaluated by two radiologists experienced in chest trauma imaging. For each patient, the following findings were recorded: number of rib fractures, laterality, anatomical distribution, degree of displacement, and associated chest injuries, including pulmonary contusion, hemothorax, pneumothorax, subcutaneous emphysema, and atelectasis. Disagreements were resolved by joint reassessment and consensus.

RibScore was calculated according to the original definition [10] and consisted of the following components: ≥ 6 rib fractures, bilateral rib fractures, flail chest, at least three significantly displaced rib fractures, first rib fracture, and rib fractures involving all three anatomical regions (anterior, lateral, and posterior). Each component contributed 1 point, yielding a total score ranging from 0 to 6.

### 2.4 Outcome Definition

The primary outcome was new‐onset pneumonia during hospitalization. Pneumonia was defined as the presence of new or progressive pulmonary infiltrates on chest imaging 48 h after admission, combined with at least two of the following clinical or laboratory criteria: body temperature > 38°C or < 36°C; white blood cell count > 10 × 10^9^/L or < 4 × 10^9^/L; new or increased purulent respiratory secretions; cough, dyspnea, or worsening oxygenation relative to baseline; or positive respiratory pathogen test results suggestive of infection.

Pneumonia was diagnosed by the attending physicians based on clinical manifestations, laboratory findings, changes in chest imaging, and microbiological data, and was subsequently verified by the research team using the predefined criteria. Preexisting pulmonary infection at admission was not considered new‐onset in‐hospital pneumonia. For mechanically ventilated patients, pneumonia fulfilling the same diagnostic criteria during hospitalization was also included as the primary outcome.

### 2.5 Candidate Variable Selection and Model Development

Candidate predictors were prespecified based on the literature, clinical availability, and their potential association with post‐traumatic pulmonary infection. Univariate logistic regression was first used to screen for variables associated with in‐hospital pneumonia (*p* < 0.10 and clinically meaningful); those meeting the criteria were entered into least absolute shrinkage and selection operator (LASSO) regression. Prior to LASSO analysis, continuous variables were standardized. The optimal penalty parameter λ was determined via 10‐fold cross‐validation, and the non‐zero coefficient variables at λ1SE (the largest λ within one standard error of the minimum) were selected to form a parsimonious candidate set. Further selection was then performed based on clinical interpretability, inter‐variable correlations, and avoidance of highly overlapping indicators that reflect the same injury dimension (e.g., number of rib fractures, RibScore, AIS, and ISS). A multivariate logistic regression model was ultimately established using in‐hospital pneumonia as the dependent variable, and the predicted probability of pneumonia was calculated for each patient.

### 2.6 Model Evaluation and Statistical Analysis

Discriminative ability was assessed using receiver operating characteristic (ROC) curves, and the area under the curve (AUC) with its 95% confidence interval (CI) was calculated. The optimal cutoff value was determined using the Youden index, and the corresponding sensitivity and specificity were obtained. The DeLong test was used to compare the AUCs of the model and RibScore, the latter serving as an imaging‐based reference. RibScore was used solely for comparative purposes and was not incorporated into model development. For this comparison, predicted probabilities were obtained from a separate univariable logistic regression model with RibScore as the sole predictor; therefore, the reported optimal cutoff for RibScore was expressed on the predicted‐probability scale. Calibration was evaluated using calibration plots, and the Brier score, calibration intercept, and calibration slope were calculated. Internal validation was performed using bootstrap resampling with 200 iterations to estimate optimism and derive a bias‐corrected AUC. Given the limited sample size, simple random data splitting was not conducted.

Decision curve analysis (DCA) was performed to evaluate the clinical net benefit of the model across a range of threshold probabilities, with comparisons against the “treat all” and “treat none” strategies. Patients were categorized into low‐, intermediate‐, and high‐risk groups based on tertiles of the predicted probability, and pneumonia incidence was compared across groups to evaluate risk stratification.

Continuous variables were summarized as mean ± SD or median (IQR) according to their distribution, and between‐group comparisons were made using *t* test or Mann–Whitney *U* test. Categorical variables were reported as frequencies (percentages) and compared using the chi‐square test or Fisher's exact test. Logistic regression results were reported as odds ratios (OR) with 95% CIs. A two‐sided *p* < 0.05 was considered statistically significant. All analyses used R version 4.4.3, with the *glmnet* and *pROC* packages.

## 3 Results

### 3.1 Patients With In‐Hospital Pneumonia Were Older, Had More Hypoxia, and Greater Chest Injury Burden

A total of 217 adult patients with blunt chest trauma and rib fractures were enrolled; 53 (24.4%) developed in‐hospital pneumonia, while the remaining 164 did not.

Compared with the non‐pneumonia group, patients who developed pneumonia were older (mean age [67.2 ± 15.9] years vs. [56.7 ± 14.6] years, *p* < 0.001), had lower SpO_2_ on admission ([94.7 ± 2.8]% vs. [96.0 ± 2.1]%, *p* = 0.003), higher white blood cell counts ([12.9 ± 3.7] × 10^9^/L vs. [10.7 ± 3.5] × 10^9^/L, *p* < 0.001), more rib fractures (5.0 [4.0–6.0] vs. 4.0 [3.0–5.0], *p* < 0.001), and a higher NLR (7.0 [4.4–10.5] vs. 5.2 [3.4–7.3], *p* = 0.003). The proportion of patients with SpO_2_ < 95% was significantly higher in the pneumonia group than in the non‐pneumonia group (47.2% vs. 21.3%, *p* < 0.001), as were the proportions with ≥ 6 rib fractures (37.7% vs. 19.5%, *p* = 0.007), pulmonary contusion (62.3% vs. 29.3%, *p* < 0.001), hemothorax (45.3% vs. 23.2%, *p* = 0.002), and AIS ≥ 3 in other body regions (35.8% vs. 15.2%, *p* = 0.001). No significant differences were observed between the two groups with respect to sex, chronic obstructive pulmonary disease (COPD), pain Numeric Rating Scale (NRS) score, pneumothorax, or subcutaneous emphysema (all *p* > 0.05). Detailed baseline characteristics are presented in Table 1.

**TABLE 1:** Baseline characteristics of the study population according to in‐hospital pneumonia status.

Characteristic	Overall (*n* = 217)	Non‐pneumonia (*n* = 164)	Pneumonia (*n* = 53)	*p*
Age, years	59.3 ± 15.6	56.7 ± 14.6	67.2 ± 15.9	< 0.001
Male sex	140 (64.5%)	102 (62.2%)	38 (71.7%)	0.209
COPD	26 (12.0%)	17 (10.4%)	9 (17.0%)	0.197
SpO_2_, %	95.7 ± 2.4	96.0 ± 2.1	94.7 ± 2.8	0.003
SpO_2_ < 95%	60 (27.6%)	35 (21.3%)	25 (47.2%)	< 0.001
WBC, ×10^9^/L	11.2 ± 3.7	10.7 ± 3.5	12.9 ± 3.7	< 0.001
NLR	5.6 (3.5–8.5)	5.2 (3.4–7.3)	7.0 (4.4–10.5)	0.003
CRP, mg/L	29.7 (12.2–49.5)	29.0 (12.6–46.9)	43.3 (11.1–61.4)	0.051
Albumin, g/L	38.0 ± 4.4	38.3 ± 4.4	37.1 ± 4.2	0.099
Number of fractured ribs	4.0 (3.0–5.0)	4.0 (3.0–5.0)	5.0 (4.0–6.0)	< 0.001
Rib fractures ≥ 6	52 (24.0%)	32 (19.5%)	20 (37.7%)	0.007
ISS	16.0 (12.0–21.0)	16.0 (12.0–20.0)	17.0 (11.0–24.0)	0.079
Pain NRS	5.0 (4.0–7.0)	5.0 (4.0–7.0)	5.0 (4.0–8.0)	0.555
Lung contusion	81 (37.3%)	48 (29.3%)	33 (62.3%)	< 0.001
Hemothorax	62 (28.6%)	38 (23.2%)	24 (45.3%)	0.002
Pneumothorax	63 (29.0%)	45 (27.4%)	18 (34.0%)	0.363
Subcutaneous emphysema	38 (17.5%)	27 (16.5%)	11 (20.8%)	0.475
Other‐system AIS ≥ 3	44 (20.3%)	25 (15.2%)	19 (35.8%)	0.001
ICU admission within 24 h	38 (17.5%)	23 (14.0%)	15 (28.3%)	0.017
Mechanical ventilation within 24 h	16 (7.4%)	9 (5.5%)	7 (13.2%)	0.073

*Note:* Data are presented as mean ± SD, median (IQR), or *n* (%), as appropriate. Normally distributed continuous variables were compared using the independent‐samples *t* test; non‐normally distributed variables were compared using the Mann–Whitney *U* test. Categorical variables were compared using the chi‐square test or Fisher's exact test, as appropriate.

Abbreviations: AIS, Abbreviated Injury Scale; COPD, chronic obstructive pulmonary disease; CRP, C‐reactive protein; ICU, intensive care unit; IQR, interquartile range; ISS, Injury Severity Score; NLR, neutrophil‐to‐lymphocyte ratio; NRS, Numeric Rating Scale; SpO_2_, peripheral oxygen saturation; WBC, white blood cell count.

### 3.2 Five Early Clinical Features Were Independent Predictors of In‐Hospital Pneumonia

Variables with a univariate *p* < 0.10 and clinical relevance were entered into the LASSO regression. At λ1SE, LASSO retained 11 candidate variables: pulmonary contusion, SpO_2_ < 95%, hemothorax, AIS ≥ 3 in other body regions, chest AIS, RibScore, age, number of rib fractures, white blood cell count, NLR, and CRP (Figure 1A,B).

**FIGURE 1:**
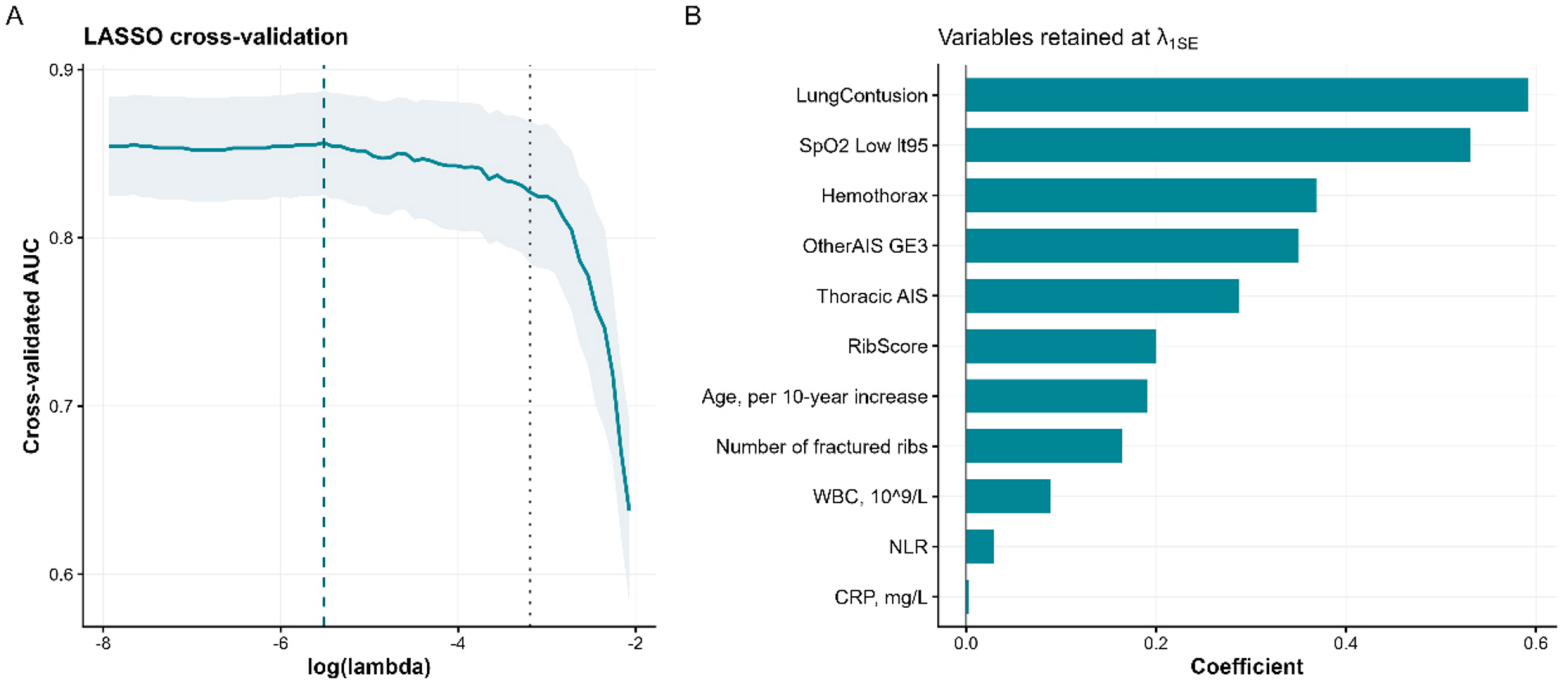
LASSO‐based predictor selection for in‐hospital pneumonia. (A) Ten‐fold cross‐validation curve showing the cross‐validated area under the ROC curve across log‐transformed penalty parameters. The dashed and dotted vertical lines indicate λmin and λ1SE, respectively. (B) Predictors retained at λ1SE and their corresponding non‐zero coefficients. Abbreviations: AUC, area under the receiver operating characteristic curve; CRP, C‐reactive protein; NLR, neutrophil‐to‐lymphocyte ratio; ROC, receiver operating characteristic; SpO_2_, peripheral oxygen saturation; WBC, white blood cell count.

Because some candidate indicators overlapped in reflecting chest injury burden or inflammation, and to control complexity and overfitting, the final multivariate model included age, SpO_2_ < 95%, pulmonary contusion, hemothorax, and AIS ≥ 3 in other body regions. These variables were selected based on clinical representativeness, avoidance of inter‐variable redundancy, early availability, and the number of outcome events.

Multivariate logistic regression analysis showed that each 10‐year increase in age was associated with a 47.2% higher risk of in‐hospital pneumonia (OR = 1.472, 95% CI: 1.148–1.920, *p* = 0.003). SpO_2_ < 95% (OR = 3.230, 95% CI: 1.523–6.939, *p* = 0.002), pulmonary contusion (OR = 3.807, 95% CI: 1.848–8.083, *p* < 0.001), hemothorax (OR = 2.921, 95% CI: 1.380–6.279, *p* = 0.005), and AIS ≥ 3 in other body regions (OR = 2.503, 95% CI: 1.081–5.766, *p* = 0.031) were each independently associated with an increased risk of in‐hospital pneumonia. Univariate and multivariate regression results for the final model variables are summarized in Table 2.

**TABLE 2:** Univariable and multivariable logistic regression analyses of factors associated with in‐hospital pneumonia.

Predictor	Univariable OR	95% CI	*p*	Multivariable OR	95% CI	*p*
Age, per 10‐year increase	1.606	1.290–2.031	< 0.001	1.472	1.148–1.920	0.003
SpO_2_ < 95%	3.291	1.708–6.371	< 0.001	3.230	1.523–6.939	0.002
Lung contusion	3.988	2.102–7.740	< 0.001	3.807	1.848–8.083	< 0.001
Hemothorax	2.744	1.428–5.277	0.002	2.921	1.380–6.279	0.005
Other‐system AIS ≥ 3	3.107	1.528–6.294	0.002	2.503	1.081–5.766	0.031
Thoracic AIS	2.108	1.334–3.395	0.002	—	—	—
RibScore	2.122	1.486–3.094	< 0.001	—	—	—
Number of fractured ribs	1.510	1.240–1.864	< 0.001	—	—	—
WBC, per 1 × 10^9^/L increase	1.189	1.088–1.308	< 0.001	—	—	—
NLR, per 1‐unit increase	1.072	1.025–1.126	0.003	—	—	—
CRP, per 1 mg/L increase	1.016	1.003–1.029	0.014	—	—	—

*Note:* Univariable estimates are shown for all variables selected by LASSO at λ1SE; multivariable estimates are shown only for the five variables retained in the final model. A dash (—) indicates that the variable was not included in the multivariable model.

Abbreviations: AIS, Abbreviated Injury Scale; CI, confidence interval; CRP, C‐reactive protein; LASSO, least absolute shrinkage and selection operator; NLR, neutrophil‐to‐lymphocyte ratio; OR, odds ratio; SpO_2_, peripheral oxygen saturation; WBC, white blood cell count.

### 3.3 The Five‐Variable Model Showed Good Discriminative Ability for In‐Hospital Pneumonia

The final model exhibited good discriminative ability for in‐hospital pneumonia, with an AUC of 0.813 (95% CI: 0.744–0.883). At the optimal cutoff value of 0.227, the model achieved a sensitivity of 75.5%, a specificity of 75.0%, and an accuracy of 75.1%.

As an imaging‐based reference for injury severity, RibScore yielded an AUC of 0.678 (95% CI: 0.596–0.761) for predicting in‐hospital pneumonia. At its optimal cutoff of 0.301, the sensitivity was 49.1%, specificity 82.9%, and accuracy 74.7%. The DeLong test showed that the final model had a significantly higher AUC than RibScore (0.813 vs. 0.678, *p* = 0.009), indicating that integration of age, early hypoxia, chest injury characteristics, and severe injury in other body regions provided superior discriminative performance compared with a score based solely on rib fracture patterns (Table 3; Figure 2A).

**TABLE 3:** Discriminative performance of the final model and RibScore for predicting in‐hospital pneumonia.

Performance metric	Final model	RibScore	*p*
AUC (95% CI)	0.813 (0.744–0.883)	0.678 (0.596–0.761)	0.009
Optimal threshold	0.227	0.301	—
Sensitivity	75.5%	49.1%	—
Specificity	75.0%	82.9%	—
Accuracy	75.1%	74.7%	—

*Note:* The *p* value was calculated using the DeLong test for comparison of AUCs between the final model and RibScore. For both models, the optimal threshold is the predicted probability cutoff determined by the Youden index. RibScore predicted probabilities were obtained from a separate univariable logistic regression; its threshold of 0.301 corresponds to a RibScore of at least 2. Sensitivity, specificity, and accuracy were calculated at the respective thresholds.

Abbreviations: AUC, area under the receiver operating characteristic curve; CI, confidence interval.

**FIGURE 2:**
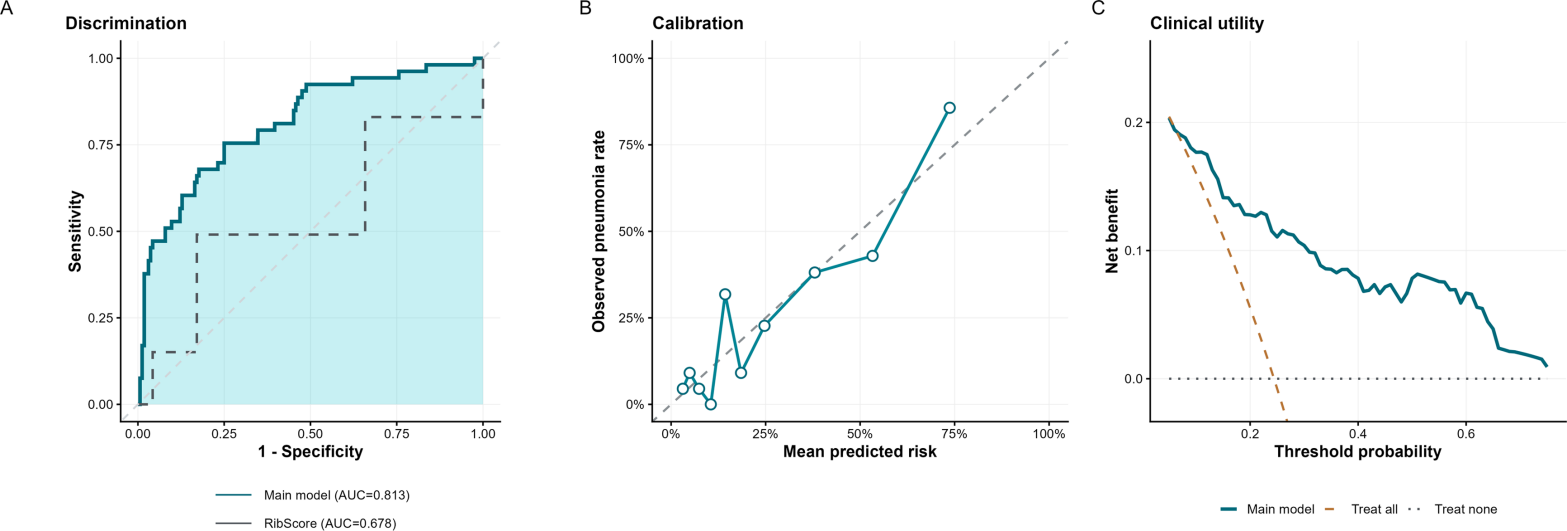
Performance of the final early‐pneumonia prediction model. (A) ROC curves for the final model and RibScore. The shaded area represents the area under the receiver operating characteristic curve of the final model. (B) Calibration plot comparing the observed incidence of in‐hospital pneumonia with the mean predicted probability. The diagonal line represents perfect calibration. (C) Decision curve analysis showing the net benefit of the final model across a range of threshold probabilities, compared with the treat‐all and treat‐none strategies. Abbreviations: AUC, area under the receiver operating characteristic curve; ROC, receiver operating characteristic.

### 3.4 Model Performance Remained Stable After Internal Validation

The final model achieved a Brier score of 0.133. Apparent calibration based on the derivation cohort revealed a calibration intercept of 0.000 and a calibration slope of 1.000. The calibration plot showed general agreement between predicted probabilities and observed pneumonia incidence, although some deviations were observed in specific risk ranges (Figure 2B).

Internal validation was conducted using 200 bootstrap resamples. The apparent AUC of the model was 0.813, with a mean optimism of 0.014, resulting in a bias‐corrected AUC of 0.799. The corrected AUC was only marginally lower than the apparent AUC, suggesting that the model retained satisfactory discriminative ability after adjustment for potential overfitting.

Decision curve analysis showed that the model provided positive net benefit across the examined threshold probabilities and outperformed both treat‐all and treat‐none strategies over a wide range, supporting its potential clinical utility (Figure 2C).

### 3.5 The Model Identified High‐Risk Populations With Pneumonia Incidence Increasing From 5.5% to 50.0%

Patients were stratified into low‐, intermediate‐, and high‐risk groups according to tertiles of the predicted probability derived from the final model, comprising 73, 72, and 72 patients, respectively. The median predicted risks in the three groups were 5.2%, 16.1%, and 49.0%, respectively.

Pneumonia occurred in 4, 13, and 36 patients in the low‐, intermediate‐, and high‐risk groups, corresponding to incidences of 5.5%, 18.1%, and 50.0%, respectively, demonstrating a clear gradient across risk strata (Table 4; Figure 3A). The rates of intensive care unit (ICU) admission during hospitalization were 17.8%, 18.1%, and 26.4% in the low‐, intermediate‐, and high‐risk groups, respectively, and the corresponding rates of mechanical ventilation were 6.8%, 8.3%, and 11.1%.

**TABLE 4:** Risk stratification and outcomes according to the final prediction model.

Outcome	Low risk (*n* = 73)	Intermediate risk (*n* = 72)	High risk (*n* = 72)
Number of patients	73	72	72
Pneumonia, *n* (%)	4 (5.5%)	13 (18.1%)	36 (50.0%)
Median predicted risk	5.2%	16.1%	49.0%
ICU admission during hospitalization	17.8%	18.1%	26.4%
Mechanical ventilation during hospitalization	6.8%	8.3%	11.1%

*Note:* Risk groups were defined by tertiles of the predicted probability from the final model. ICU admission and mechanical ventilation were assessed over the entire hospitalization. Percentages in parentheses for pneumonia are based on the group size. *p* for trend across groups for pneumonia incidence was < 0.001 (not shown).

Abbreviation: ICU, intensive care unit.

**FIGURE 3:**
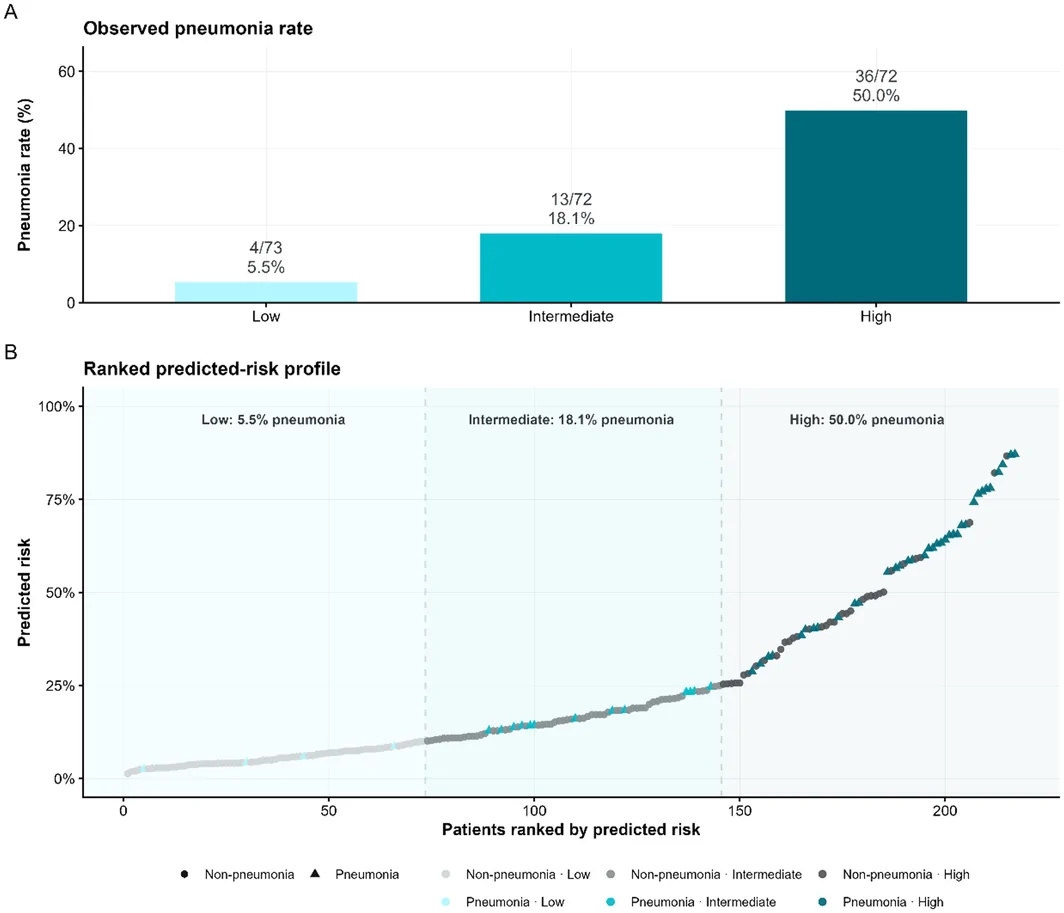
Risk stratification according to the final prediction model. (A) Observed pneumonia rates across the low‐, intermediate‐, and high‐risk groups defined by tertiles of the predicted probability. (B) Ranked individual predicted‐risk profile. Patients were ordered from the lowest to the highest predicted probability; marker shape indicates pneumonia status, and color intensity indicates the model‐defined risk group. Abbreviation: ICU, intensive care unit.

When individual predicted probabilities were ordered from lowest to highest, patients with pneumonia were progressively concentrated in the higher‐risk regions, with marked enrichment of pneumonia cases in the high‐risk group and relatively few cases in the low‐risk group (Figure 3B). These findings indicate that the model not only quantifies individual pneumonia risk but also stratifies patients into subgroups with substantially different probabilities.

## 4 Discussion

This study developed a risk prediction model for in‐hospital pneumonia using early admission data from adult patients with blunt chest trauma and rib fractures. LASSO at λ1SE retained 11 variables, which were refined to five predictors—age, SpO_2_ < 95%, pulmonary contusion, hemothorax, and AIS ≥ 3 in other body regions—based on clinical representativeness, early availability, and avoidance of redundancy. The final model captured multiple dimensions including host susceptibility, early respiratory physiologic status, local chest injury, and overall trauma burden, and demonstrated good discriminative ability with only modest performance degradation after bootstrap correction. Risk stratification by predicted probability tertiles yielded a clear gradient in pneumonia incidence across low‐, intermediate‐, and high‐risk groups. These findings suggest that in‐hospital pneumonia risk is not determined by rib fracture number or morphology alone, but by a combination of diverse early clinical and injury‐related factors.

The incidence of in‐hospital pneumonia in this cohort was 24.4%. Previous studies have reported widely varying pneumonia rates, likely due to differences in age, fracture burden, polytrauma prevalence, diagnostic criteria, and observation periods. Prior research in patients with multiple rib fractures identified age, injury severity, and rib fracture burden as potential contributors to pneumonia development [17]. Subsequent studies focusing on rib fracture patients have suggested that advanced age, greater chest injury severity, and preexisting pulmonary disease are associated with elevated pneumonia risk [18]. In broader trauma populations, hospital‐acquired pneumonia is additionally influenced by early physiologic derangements, trauma burden, and therapeutic interventions [19]. Therefore, our observed incidence should be interpreted as the pneumonia burden in a hospitalized blunt chest trauma cohort and should not be directly compared with that in low‐risk, isolated, or outpatient‐managed rib fracture populations.

Age emerged as an independent predictor in the final model. With advancing age, chest wall compliance, respiratory muscle reserve, and effective cough capacity tend to decline, predisposing patients to shallow breathing, secretion retention, and atelectasis in the setting of pain and restricted chest wall motion. Previous studies of elderly blunt trauma patients have shown that the risk of adverse outcomes increases markedly once the number of rib fractures reaches a certain threshold [20]. Geriatric rib fracture management models have also included age and early injury characteristics as important risk indicators [21]. During candidate variable selection, age, number of rib fractures, and RibScore were all considered; ultimately age was retained while multiple fracture‐burden indicators were excluded to reduce redundancy. This suggests that age may capture risk beyond fracture burden, reflecting overall physiological tolerance to trauma and respiratory impairment.

SpO_2_ < 95% was a key early physiological predictor in the model. Hypoxemia may reflect gas exchange impairment resulting from pulmonary contusion, hemothorax, atelectasis, painful hypoventilation, or other coexisting injuries. Previous studies have shown that combining admission physiology with imaging improves identification of patients at risk for pulmonary complications and aids management [16]. Deterioration in dynamic physiologic indices—such as respiratory rate, pain, and ventilatory function—has also been associated with adverse outcomes including pneumonia and high oxygen requirements, highlighting the prognostic importance of early changes in gas exchange and mechanics [22]. Of note, hypoxemia itself should not be construed as a direct cause of pneumonia, but rather as an integrated marker of early respiratory compromise and underlying pulmonary injury severity.

Both pulmonary contusion and hemothorax were independently associated with in‐hospital pneumonia. Pulmonary contusion can impair the alveolar‐capillary barrier, cause interstitial and alveolar edema, create ventilation‐perfusion mismatch, and amplify local inflammation, thereby reducing pulmonary reserve and impairing secretion clearance. Previous models based on contusion severity have shown that pulmonary parenchymal injury information can assess subsequent pulmonary complication risk [23]. Quantitative assessments of chest wall injury have also suggested that the combined burden of chest wall and lung injury is closely related to pneumonia [24]. Hemothorax may increase susceptibility to pulmonary infection through compression of lung tissue, restriction of ventilation, promotion of atelectasis, and as a marker of higher‐energy chest injury. Earlier work has shown that rib fracture burden and severity of lung, head, and abdominal injuries are independently associated with adverse outcomes in trauma patients [25]. Thus, the role of hemothorax in this model likely encompasses both direct respiratory physiological effects and broader indications of thoracic injury severity.

The inclusion of AIS ≥ 3 in other body regions in the final model indicates that in‐hospital pneumonia risk cannot be fully explained by local chest injury alone. Severe extrathoracic injuries may be accompanied by decreased consciousness, restricted mobility, surgical interventions, transfusion requirements, sedation and analgesia, and increased respiratory support, all of which can affect sputum clearance, lung re‐expansion, and infection susceptibility. In polytrauma patients, the performance of chest trauma scores may vary by overall injury context, and different systems have shown variable performance in isolated chest injury versus polytrauma cohorts [26]. Studies on early suspected ventilator‐associated pneumonia in severe blunt chest trauma have also indicated that infection risk is related to overall trauma burden and treatment exposure, not chest imaging alone [27]. Retaining AIS ≥ 3 in other body regions helps the model capture additional risk information from multisystem injuries.

Not all candidate variables retained at λ1SE were incorporated into the conventional multivariate logistic regression. This decision was made because thoracic AIS, RibScore, number of rib fractures, pulmonary contusion, and hemothorax all reflect overlapping aspects of chest injury burden, whereas WBC, NLR, and CRP represent post‐traumatic inflammatory or stress responses. Including all these highly correlated or redundant indicators in a model with only 53 pneumonia events would increase the risk of parameter instability and overfitting. The final five variables each represent distinct clinical dimensions; thus, parsimony was based not on univariate *p*‐values alone but on statistical stability, clinical interpretability, and implementability. Notably, WBC is also one of the laboratory criteria used in pneumonia diagnosis, and to reduce potential overlap between predictors and the outcome definition, it was excluded from the final model.

Compared with some complex machine learning or nomogram models, the final model retained only five early clinical variables. Machine learning has been used to predict in‐hospital pneumonia in flail chest patients, suggesting that multivariable algorithms may improve identification of high‐risk individuals [28]. Recent artificial intelligence models for chest trauma have also shown promise in predicting pulmonary complications [29]. Moreover, a nomogram specifically for pneumonia in routinely managed rib fracture patients further illustrates the need for outcome‐specific tools [30]. However, increased model complexity does not necessarily translate into better generalizability. In contrast, the model's variables are all routinely available early, offering good interpretability and ease of use, making it suitable for bedside initial risk stratification.

Beyond discrimination, this study also evaluated calibration, internal validation, and clinical net benefit. The model achieved a Brier score of 0.133, and apparent calibration was generally acceptable. After bootstrap correction, the AUC decreased from 0.813 to 0.799, with a mean optimism of 0.014, suggesting limited overfitting. Risk stratification by predicted probability tertiles yielded pneumonia incidences from 5.5% in the low‐risk group to 50.0% in the high‐risk group, demonstrating that the model can generate clinically meaningful risk categories. This stratification may support closer oxygenation monitoring, analgesia optimization, lung recruitment, breathing exercises, secretion management, and monitoring intensity decisions, but should not directly guide prophylactic antibiotics.

Several limitations should be acknowledged. First, this was a retrospective, single‐center study, and the case mix, imaging interpretation processes, and clinical management pathways may affect model performance; external generalizability requires further validation. Second, although pneumonia outcomes were verified using uniform criteria, some clinical information may have been affected by record completeness and diagnostic variability; moreover, factors such as frailty, cough effectiveness, baseline lung function, dynamic oxygenation changes, analgesic modalities, and rehabilitation intensity were not fully captured. Third, only internal validation via bootstrap was performed; temporal or independent external validation has not yet been conducted. Fourth, the risk group cutoffs were derived from tertiles in this cohort and are intended to illustrate stratification capacity, not as fixed thresholds for other centers. Future work should include external validation in independent, multicenter cohorts with different trauma care levels, and further evaluate calibration, clinical utility, and implementability.

## 5 Conclusions

Age, SpO_2_ < 95%, pulmonary contusion, hemothorax, and AIS ≥ 3 in other body regions were independent risk factors for in‐hospital pneumonia. The model based on these predictors demonstrated good discriminative ability, maintained stable performance after internal validation, and produced risk strata with substantially different pneumonia incidences. This model may be useful for early identification of high‐risk patients at admission, though further validation in independent, multicenter cohorts is needed.

## Author Contributions

Conceptualization, KaiChao Yang; data curation, WeiXi Zhong and JianYin Huang; formal analysis, YiFeng Liu and Lei Geng; investigation, WeiXi Zhong and Qing Xu; methodology, JianYin Huang, YiFeng Liu and Lei Geng; writing – original draft preparation, KaiChao Yang; writing – review and editing, Qing Xu. All authors have read and agreed to the published version of the manuscript.

## Funding

The authors have nothing to report.

## Ethics Statement

All procedures performed in this study involving human participants were in accordance with the ethical standards of the institutional and/or national research committee and with the 1964 Helsinki Declaration and its later amendments or comparable ethical standards. All subjects were approved by Shanghai Sixth People's Hospital Affiliated to Shanghai Jiao Tong University School of Medicine.

## Conflicts of Interest

The authors declare no conflicts of interest.

## Data Availability

Data are available from the corresponding author on request.
